# Identification of Prognostic Biomarkers of Glioblastoma Based on Multidatabase Integration and Its Correlation with Immune-Infiltration Cells

**DOI:** 10.1155/2022/3909030

**Published:** 2022-05-31

**Authors:** Wencong Ding, Xian Zhou, Guoqiang Jiang, Weiwei Xu, Songkai Long, Fan Xiao, Yongshi Liao, Jia Liu

**Affiliations:** ^1^Department of Neurosurgery, the Affiliated Nanhua Hospital, Hengyang Medical School, University of South China, Hengyang 421002, Hunan, China; ^2^Department of Hepatobiliary Surgery, the Affiliated Nanhua Hospital, Hengyang Medical School, University of South China, Hengyang 421002, Hunan, China; ^3^Department of Clinical Laboratory, The Affiliated Nanhua Hospital, Hengyang Medical School, University of South China, Hengyang 421002, Hunan, China; ^4^Department of Anesthesiology, Affiliated Nanhua Hospital, University of South China, Hengyang 421001, Hunan, China; ^5^Clinical Research Institute, Hunan Provincial Clinical Research Center for Metabolic Associated Fatty Liver Disease, the Affiliated Nanhua Hospital, Hengyang Medical School, University of South China, Hengyang 421002, Hunan, China

## Abstract

**Background:**

Glioblastoma (GBM) is the most malignant of all known intracranial tumors; meanwhile, most patients have a poor prognosis. In order to improve the poor prognosis of GBM patients as much as possible, it is specifically significant to identify biomarkers related to the gene diagnosis and gene therapy.

**Methods:**

In this study, a total of 343 GBM specimens and 259 nontumor specimens were collected from four Gene Expression Omnibus (GEO) datasets and The Cancer Genome Atlas (TCGA) database; then, we analyzed the differentially expressed genes (DEGs) from the above data. Through Venn diagram analysis, 54 common upregulated DEGs and 22 common downregulated DEGs were triumphantly recognized.

**Results:**

On the basis of the degree of formation communication in protein-protein interaction network (PPIN), the 10 upregulated central genes were ranked, incorporating LOX, IGFBP3, CD44, TIMP1, FN1, VEGFA, POSTN, COL1A1, COL1A2, and COL3A1. By combining the expression levels and the clinical features of GBM, we found that four hub genes (TIMP1, FN1, POSTN, and LOX) were significantly upregulated and related to poor prognosis of GBM. Meanwhile, univariate and multivariate Cox regression analysis suggested that TIMP1 could be one of the independent prognostic factors for GBM patients. Furthermore, TIMP1 was particularly correlated with the immune marker of macrophage M1, macrophage M2, neutrophils, tumor-associated macrophage, and Tregs. We then analyzed the role of TIMP1 in GBM cancer cell lines by relevant experiments, which indicated that TIMP1 knockdown resulted in the decreased cell proliferation, migration, and invasion.

**Conclusions:**

Taken together, these findings demonstrated that TIMP1 might be a new biomarker to determine prognosis and immune infiltration of GBM patients.

## 1. Introduction

Glioma, a malignant intracranial tumor, is caused by abnormal proliferation of glial cells [1]. Glioblastoma or glioblastoma multiforme is classified as WHOIV [2]. GBM is the most common primary intracranial tumor, accounting for 54% of all gliomas. Malignant glioblastoma is characterized by rapid proliferation, rapid infiltration, and late diagnosis [3]. Although many studies have clarified the potential molecular mechanism of glioma, there is no comprehensive and effective treatment regimen and the long-term survival rate of glioma patients cannot be effectively improved [4]. In order to improve the prognosis of patients with GBM, the objective of this research is to find novel diagnostic and prognostic biomarkers and possible therapeutic targets of GBM, which is expected to guide clinical treatment and provide new ideas for improving the outcome of GBM patients.

Bioinformatics is a subject developed rapidly in recent years which is relied on big data processing, and it can extract valuable information for clinical research from massive data generated by multisource experiments [5]. Chen et al. performed an integrated bioinformatics analysis of the pancreatic cancer dataset in TCGA and established a risk score model consisting of DEGs, which provided a more effective way for forecasting survival situation than AJCC stage [6]. By using a variety of bioinformatics tools, Wang et al. found that the increased PITX1 expression is correlated with worse relapse-free survival (RFS), disease-specific survival (DSS), and overall survival (OS) in breast cancer patients. Therefore, it may provide a basis for oriented drug treatment related to breast cancer [7]. Zhou et al. used software *R* to normalize the data of native GBM samples and nontumorous samples in the databases. The discriminated DEGs were used in grasping of the molecular mechanisms of GBM, and those DEGs hold promise as prognostic biomarkers for future transactions of GBM sufferers [8]. Although bioinformatics analysis has been widely used in solid tumors, there is little research on bioinformatics analysis of GBM. Therefore, in-depth studies on the composition, functional changes, and immune effects of GBM mutated genes are of positively consequence for forecasting the prognosis of GBM patients and for the exploitation of potential targeted treatment.

The tumor microenvironment (TME) is the cell environment that tumor cells depend on for survival and growth [9]. TME consists of peripheral blood vessels, extracellular matrix (ECM), nontumor cells, and a variety of biological factors that are influenced by malignant and untransformed cells within the tumor [10]. A number of cell types have been identified in TME using different cell-specific markers, including many different types of cells, such as stromal cells, fibroblasts, and various immune cells. The growth and survival of malignant tumor cells are promoted by growth factors secreted by nontumor cells in TME. At the same time, these growth factors also act as attractors to stimulate a variety of cells to migrate to TME [11]. ECM is another key component of TME in addition to special types of cells [12]. ECM not only participates in the supporting framework of TME cells but also is an abundant pivotal growth factor [13]. ECM plays a significant role in the genesis and development of tumors. In the late stage of tumor progression, ECM commonly gets out of control and becomes disorganized. Unnatural ECM can also lead to disordered behavior of TME cells and promote neovascularization and inflammatory response [14]. TME is distinguished from other normal tissues by these unique properties, and there is new evidence that microenvironment-mediated external stimulation plays a pivotal role in the survival of tumor cells [15]. Disrupting the tumor microenvironment that surrounds and infiltrates the tumor may provide a proven treatment modality for malignancy [16].

Therefore, we conducted bioinformatics studies using the Gene Expression Profile Interaction Analysis 2 (GEPIA2) database to analyze the prognostic significance of hub genes. The correlation between core genes expression and GBM immune cell infiltration was detected using Tumor Immune Estimation Resource (TIMER) database. In order to identify potential biomarkers of glioblastoma, knocking down TIMP1 in U-87 MG and U-118 MG cell lines can inhibit cell proliferation, migration, and invasion, suggesting that TIMP1 plays a carcinogenic role in glioblastoma.

## 2. Materials and Methods

### 2.1. GEO Gene Expression Data

Keywords such as “Glioblastoma,” “Homo sapiens,” and “Array expression profile” were searched in GEO database and TCGA database, and GBM mRNA expression datasets retrieved by the above keywords were screened. After a systematic review, four GSE profiles (GSE4290, GSE7696, GSE13276, and GSE29796) and one TCGA profile (glioblastoma multiforme) were selected and downloaded. The details are shown in Table 1. All data are freely available, and no human or animal experiments were involved in this study.

### 2.2. DEGs Filtering

GEO data obtained DEGs between GBM and nontumorous brain tissue by using the GEO2R tool to standardize and convert original data from previously selected datasets into log2 format, setting screening criteria for log-fold change (FC)≥2 and *P* value < 0.05 in each file. Then, the volcano diagram of DEGs was revealed by visual hierarchical clustering analysis, and the overlapping genes were inspected in Venn diagram.

### 2.3. Functional Annotation and Signaling Pathway Analysis of the Hub Genes

To reveal the functions of DEGs, an online platform for data analysis and visualization (http://www.bioinformatics.com.cn) was used to dispose the GO function and KEGG paths of common upregulated and downregulated DEGs. GO term and KEGG pathways with *P* < 0.05 were considered statistically significant; meanwhile, Adj. *P* < 0.05 was regarded as statistically significant.

### 2.4. PPI Network Analysis

We used the Search Tool for the Retrieval of Interacting Genes (STRING) [17] to process and analyze the obtained DEGs. The filtrated 54 common upregulated DEGs had beforehand been handed over to the STRING. High-degree nodes are considered to be the most significant in this network. In this experiment, we selected the 10 genes with the highest connectivity as the central genes after the statistics of Cytoscape (v3.7.1) plugin cytoHubba.

### 2.5. Detection of Hub Genes in GBM

The GEPIA2 database [18] was used to visualize the mRNA expression of screened out genes in GBM and nonmalignant brain specimens. Human Protein Atlas (HPA) was used to determine the protein expression levels of 10 HUB genes in nonmalignant brain specimens and GBM organizations [19].

### 2.6. Extract RNAseq Data and Establish Survival and Risk Curves

Using the UCSC XENA (https://xenabrowser.net/datapages/) [20] after the unified processing of RNAseq data of TCGA and GTEX, the GBM data of TCGA and corresponding nonmalignant specimen data in GTEX were extracted. After log2 transformation of RNAseq data in TPM format, expression comparison among samples was conducted and the overall capability was assessed using receiver-operating characteristic (ROC) curve and area under ROC curve (AUC). For each hub gene, we used the pROC package to institute the ROC curve, calculated the AUC value, and visualized the ROC curve with the ggplot2 package. The closer the AUC value is to 1, the better the overall performance.

### 2.7. Survival Analyses and Univariate and Multivariate Cox Analysis for Hub Genes

The relationship between overall survival (OS), disease-free survival (DFS), and core genes expressed in GBM sufferers was assessed by GEPIA2. Log-rank test results with *P* < 0.01 were statistically significant. The hub genes with independent prognostic factors were further screened by univariate and multivariate Cox regression analysis.

### 2.8. Immune Infiltration Analysis

The infiltration of multiple immune cells in GBM specimens was analyzed by tumor immune estimation resources (TIMER) [21]. TIMER2.0 was used to analyze the correlation between the expression of TIMP1 and the degree of infiltration of six kinds of immune cells, including regulatory cells and cancer-related fibroblasts.

### 2.9. Cell Culture

Human glioblastoma cell lines U-87 MG and U-118 MG were purchased from Shanghai Zhong Qiao Xin Zhou Biotechnology Co., Ltd. (China). U-87 MG and U-118 MG cells were cultured in DMEM medium supplemented with 10% fetal bovine serum (FBS; Gibco) and 1% antibiotics (100 units/mL penicillin and 100 *μ*g/mL streptomycin; Gibco) in a humidified atmosphere of 5% CO2 at 37°C.

### 2.10. Construction of Lentiviral TIMP1-shRNA Vector

Lentivirus particles for short hair RNA (shRNA) of TIMP1 were purchased from HANBIO Technology (Shanghai, China). The shRNA targeting sequence for TIMP1 (sh-TIMP1) was 5′-TTCCAGTCCCGTCACCTTGC-3′, and the scrambled RNA sequence (NC) used as negative control was 5′-TTCTCCGAACGTGTCACGT-3′. U-87 MG and U-118 MG cells were infected with sh-TIMP1 and NC lentivirus particles and cultured with medium containing puromycin for 1 week.

### 2.11. Quantitative Real-Time PCR

Total mRNA was isolated by using RNA-easy Isolation Reagent (Vazyme, China) according to the manufacturer's instructions. cDNA was prepared using the HiScript III 1st Strand cDNA Synthesis Kit (Vazyme, China). qRT-PCR was performed on the ChamQ Universal SYBR qPCR Master Mix (Vazyme, China) instrument. The primer sequences are as follows: TIMP1 (forward, 5′-CATCACTACCTGCAGTTTTGTG-3'; reverse, 5′-TGGATAAACAGGGAAACACTGT-3′); *β*-actin (forward, 5′-CCTGGCACCCAGCACAAT-3'; reverse, 5′-GGGCCGGACTCGTCATAC-3′). The 2^−ΔΔCt^ method was used to determine relative quantity of each qPCR product.

### 2.12. Western Blot

Cells were lysed by using RIPA buffer (Epizyme, China) containing protease inhibitor (AbMole, USA). The total proteins were separated by 10% SDS-PAGE and subsequently transferred to PVDF membranes (Millipore, USA). After blocking with 5% fat-free milk, incubate with primary antibodies against TIMP1 (A00561-1; Boster, China) and *β*-actin (Beyotime Biotechnology, China), respectively, using a dilution of 1 : 1000 for each antibody overnight at 4°C. After washing with TBST, secondary antibodies were introduced into the membrane for 1 h at room temperature. The interested proteins were visualized using the enhanced chemiluminescence (ECL, Epizyme), and the protein band intensity was analysed using ImageJ software.

### 2.13. CCK-8 Assay and Colony Formation Assay

For CCK-8 assay, U-87 MG and U-118 MG cells transfected with sh-TIMP1 or NC were resuspended into single-cell suspension with medium containing 10% FBS, and 200 *µ*L of cell suspension was added to a 96-well plate. CCK-8 solution was added to the plate after 1, 3, 5, or 7 days of incubation and incubated for another 4 hours. Absorbance at 450 nm was measured and recorded to construct cell growth curve. For colony formation assay, U-87 MG and U-118 MG cells transfected with sh-TIMP1 or NC were plated on six-well plate with 500 cells per plate. After two weeks of incubation, cells were fixed with methanol followed by staining with 0.1% crystal violet solution.

### 2.14. Cell Migration and Invasion Assays

The ability of cell migration and cell invasion was tested by transwell experiments. U-87 MG and U-118 MG cells transfected with sh-TIMP1 or NC were plated in transwell chambers (Corning, 8 *μ*m) without or with Matrigel-coated and cultured overnight. The cells that had moved across the membrane were fixed with methanol followed by staining with 0.1% crystal violet solution. Cells that migrated into the lower chamber were counted.

## 3. Results

### 3.1. Identified DEGs

**Figure 1:**
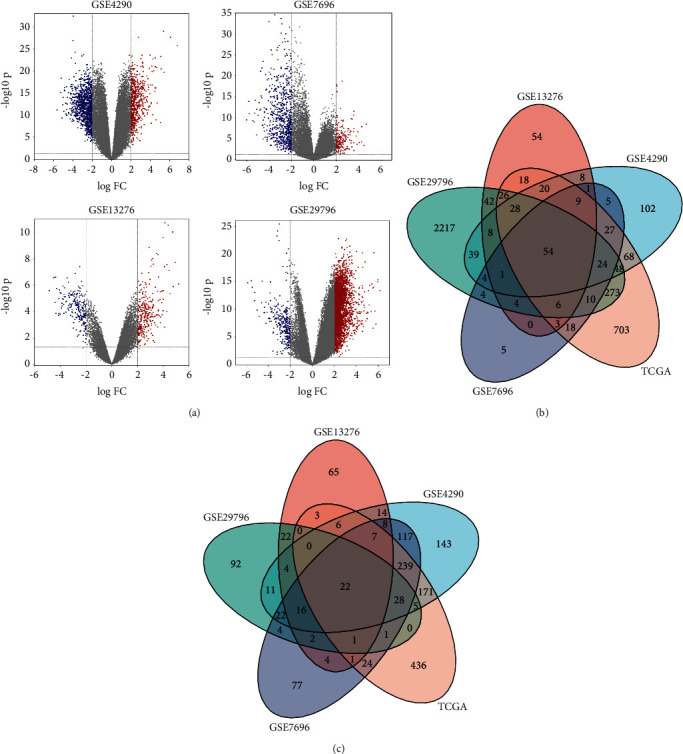
DEGs identification in different datasets of GBM. (a) Volcano diagrams of DEGs from the GBM vs. normal tissues in different datasets. |Log2FC| > 2.0, *P* value < 0.05. (b) The common upregulated genes in GSE4290, GSE7696, GSE13276, and GSE29796 and GEPIA datasets were determined by using Venn diagram. (c) The common downregulated genes in GSE4290, GSE7696, GSE13276, and GSE29796 and GEPIA datasets were determined by using Venn diagram.

The DEGs in the GSE4290, GSE7696, GSE13276, GSE29796, and TCGA datasets were identified by GEO2R. Add up to 1756 DEGs were recognized in the GSE4290, add up to 938 DEGs were identified in the GSE7696, add up to 546 DEGs were recognized in the GSE13276, add up to 3873 DEGs were recognized in the GSE29796, and add up to 2283 DEGs were recognized in TCGA (GBM). Through visual hierarchical clustering analysis, the volcano map of these DEGs is clearly shown (Figure 1(a)), where the scarlet and blue dots, respectively, represent upregulated and downregulated genes. Then, the Venn plot of the coexpression genes containing overlapping DEGs in the GSE4290, GSE7696, GSE13276, GSE29796, and TCGA datasets was constructed (Figures 1(b) and 1(c)). These 54 upregulated DEGs and 22 downregulated DEGs are plotted in Table 2. A total of 54 overlapping upregulated genes were acquired as core genes for further analysis.

### 3.2. GO and KEGG Enrichment Analyses

**Figure 2:**
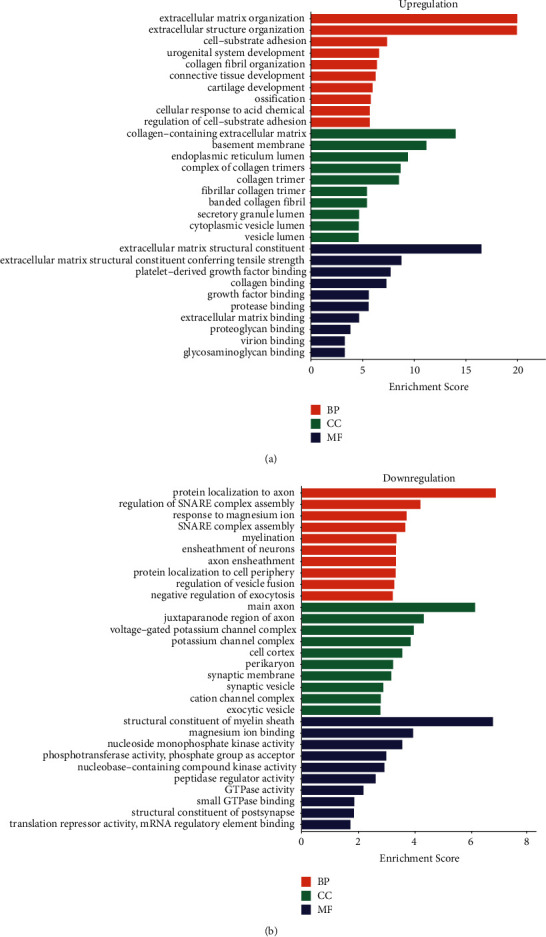
Bioinformatics analyses of DEGs in GBM. (a) GO functions of the common upregulated DEGs in GBM. (b) GO functions of the common downregulated DEGs in GBM.

**Figure 3:**
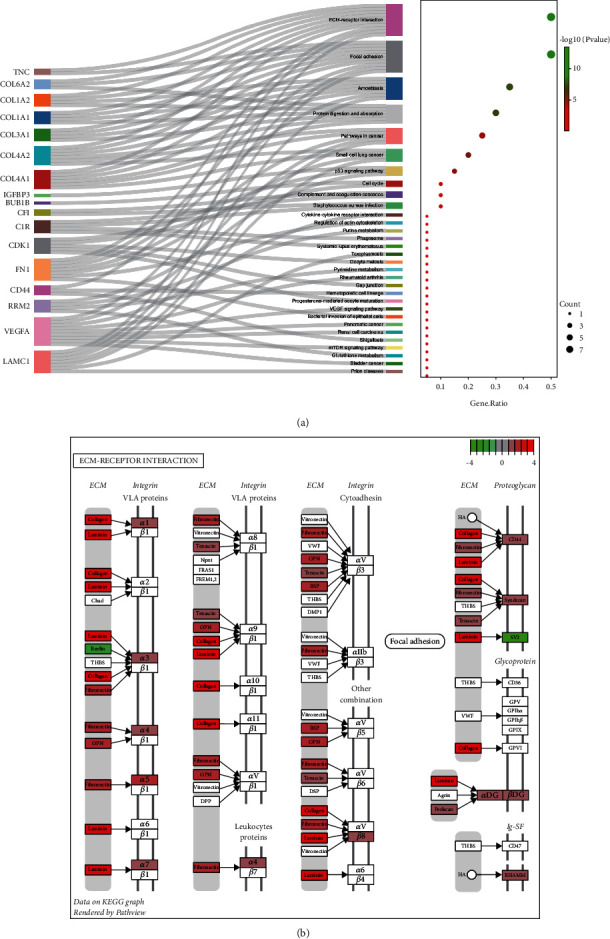
KEGG pathway enrichment analyses of DEGs in GBM. (a) 54 upregulated DEGs enrichment pathways. (b) The top enrichment pathways, ECM-receptor interaction.

We conducted GO and KEGG enrichment analyses of 54 upregulated DEGs and 22 downregulated DEGs to systematically understand the biological roles of these DEGs.  Figure 2, respectively, lists the top enriched GO terms, and Figure 3(a) lists the top 30 KEGG pathways.

GO BP displayed that 54 upregulated DEGs were obviously enriched in the lectin pathway of extracellular matrix organization, extracellular structure organization, cell-substrate adhesion, and urogenital system development. GO CC analysis showed that the most enriched terms were collagen-containing extracellular matrix, basement membrane, endoplasmic reticulum lumen, and complex of collagen trimers. The top four significantly enriched MF terms included extracellular matrix structural constituent, extracellular matrix structural constituent conferring tensile strength, platelet-derived growth factor binding, and collagen binding (Figure 2(a)).

GO BP displayed that 22 downregulated DEGs were obviously enriched in the protein localization to axon, regulation of SNARE complex assembly, and response to magnesium ion and SNARE complex assembly. GO CC analysis showed that the top obviously enriched terms were main axon, juxtaparanode region of axon, voltage-gated potassium channel complex, and potassium channel complex. The top four significantly enriched MF terms included structural constituent of myelin sheath, magnesium ion binding, nucleoside monophosphate kinase activity, and phosphotransferase activity (Figure 2(b)).

In addition, the four obvious enrichment pathways of these 54 upregulated DEGs which attracted our attention were ECM-receptor interaction, focal adhesion, pathways in cancer, and the p53 signaling pathway (Figure 3(a)). The pathway diagram shows ECM-receptor interaction is one of the top enrichment pathways (Figure 3(b)).

### 3.3. Module Screening from the PPI Network

**Figure 4:**
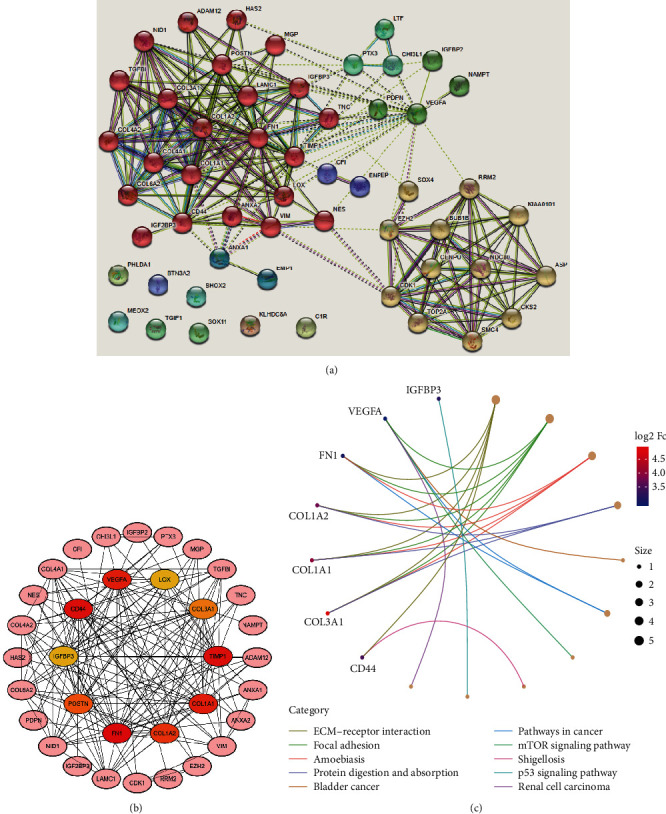
PPI network and hub genes' identification. (a) PPI network was structured by the 54 upregulated DEGs using STRING database. (b) The top 10 hub genes in the PPI network were screened ground on their connectivity degree. The genes such as CD44, COL1A1, COL1A2, COL3A1, FN1, IGFBP3, LOX, POSTN, TIMP1, and VEGFA are represented from red (high degree) to yellow (low degree). (c) Gene Ontology (GO) chord diagram of the top genes in GBM.

The PPI pairs in the 54 upregulated DEGs were determined by using the STRING (Figure 4(a)). Cytoscape identified the top 10 central genes for connectivity of the degree score (Table 3). Cytoscape also identified the most tightly connected modules (Figure 4(b)). KEGG analysis revealed that the 10 hub genes were significantly enriched in the pathways of ECM-receptor interaction, focal adhesion, amoebiasis, protein digestion and absorption, bladder cancer, pathways in cancer, mTOR signaling pathway, shigellosis, and the p53 signaling pathway (Figure 4(c)).

### 3.4. Validation of mRNA Expression in GBM

**Figure 5:**
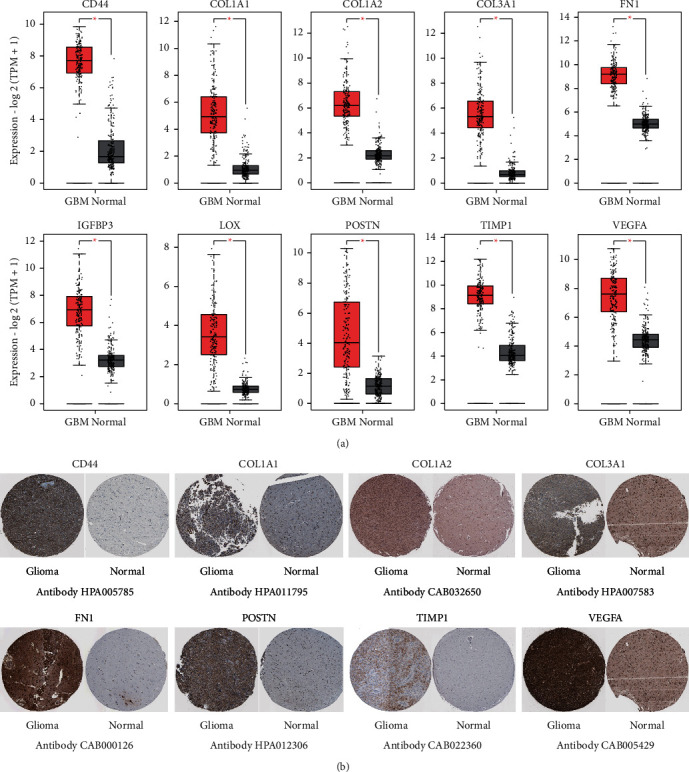
Validation of mRNA expression of the top 10 hub genes in GBM. (a) The top genes' expression levels in GBM (from TCGA database) and nonmalignant pancreas (from GTEx database) were analyzed by GEPIA (^*∗*^*P* < 0.01). (b) Representative immunohistochemistry images of the genes in GBM and nonmalignant cerebral cortex specimens from the HPA.

The results of GEPIA database displayed that the mRNA expression levels of 10 genes in GBM tissues were expressively higher than those in nonmalignant cerebral cortex specimens (*P* < 0.01) (Figure 5(a)). Notably, the protein levels of CD44, COL1A1, COL1A2, COL3A1, FN1, POSTN, TIMP1, and VEGFA were not voiced in normal cerebral cortex tissues, nevertheless, these genes moderately and highly expressed in GBM tissues (Figure 5(b)). Taken together, the consequences of this study manifested that the hub genes were overexpressed at both transcriptional and translational expression levels in GBM patients.

### 3.5. Changes in Hub Gene Frequency and Prognostic Value in GBM

**Figure 6:**
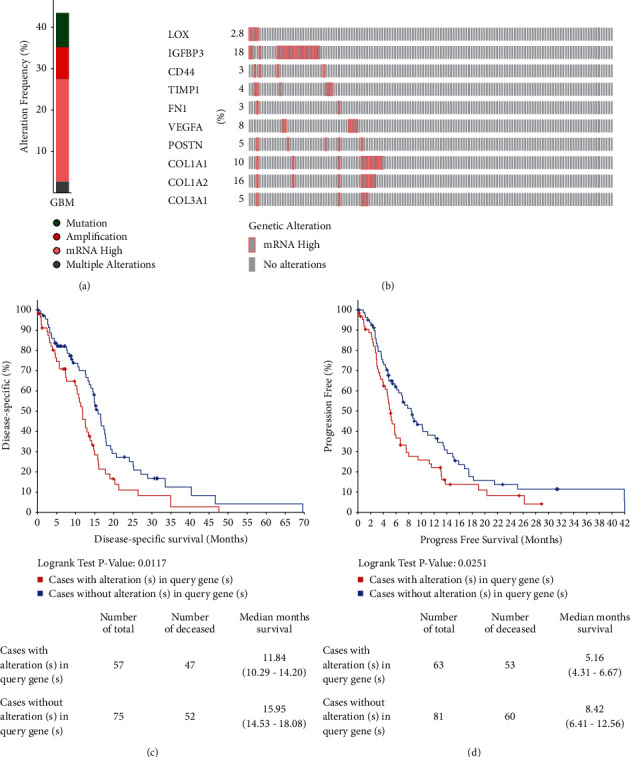
Changes in hub gene frequency and prognostic value in GBM. (a) The cBioPortal was used to calculate the percent-ages of GBM sufferers with the 10 altered hub genes. (b) mRNA expression alterations of the 10 hub genes in GBM sufferers. (c) DFS and (d) PFS of GBM patients with altered (red) and unaltered (blue) mRNA expression of 10 central genes.

The CBioPortal database was used to assess the frequency of genetic changes in these selected central genes from GBM. Approximately 43.45% of GBM clinical patients displayed vital changes in these central genes (Figure 6(a)). The mRNA alteration was the noticeably vital factors for the altered hub genes in 36 patients (24.83%) of GBM. The results showed that the percentage variation in the mRNA expression of LOX, IGFBP3, CD44, TIMP1, FN1, VEGFA, POSTN, COL1A1, COL1A2, and COL3A1 in GBM were 2.8, 18, 3, 4, 3, 8, 5, 10, 16, and 5%, respectively (Figure 6(b)).

Kaplan–Meier diagrams were used to contradistinguish DSS and progression-free survival (PFS) in GBM patients with or without changes in the mRNA expression levels of central genes. As uncovered in Figure 6(c), GBM sufferers with changed central gene expression represented notably worse DSS compared to those with unchanged central gene expression (*P* = 0.0117). Similarly, GBM sufferers with changed hub gene expression showed observably poor PFS (*P* = 0.0251) compared with those with unchanged central gene expression (Figure 6(d)).

### 3.6. Survival Curve of Hub Genes

**Figure 7:**
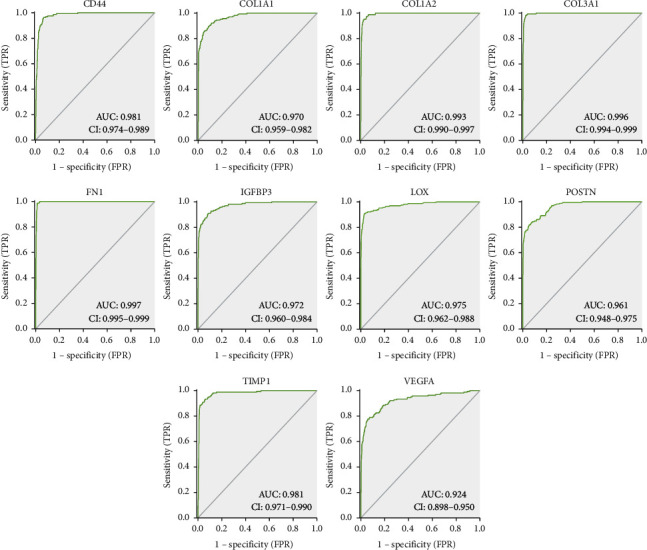
The AUC analysis of hub genes. AUC analysis of CD44, COL1A1, COL1A2, COL3A1, FN1, IGFBP3, LOX, POSTN, TIMP1, and VEGFA for distinguishing GBM samples from normal tissues.

Corresponding AUCs for these hub genes were also obtained, including CD44 (AUC = 0.981), COL1A1 (AUC = 0.970), COL1A2 (AUC = 0.993), COL3A1 (AUC = 0.996), FN1 (AUC = 0.997), IGFBP3 (AUC = 0.972), LOX (AUC = 0.975), POSTN (AUC = 0.961), TIMP1 (AUC = 0.981), and VEGFA (AUC = 0.924) (all *P* < 0.0001) (Figure 7). From the above results, it can be concluded that these variables have high accuracy in predicting the outcomes of nontumor patients and GBM patients.

### 3.7. Survival Analysis and Cox Regression Analysis in GBM

**Figure 8:**
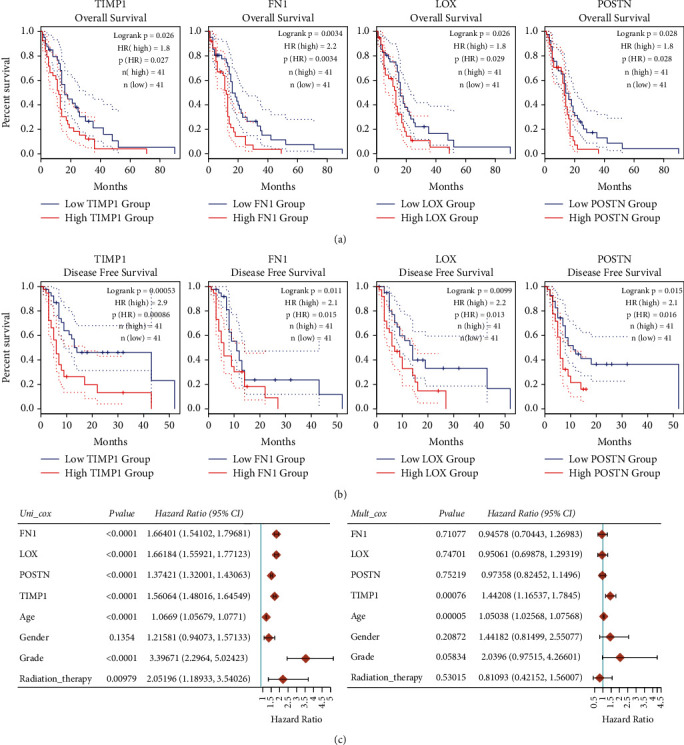
OS, DFS, and univariate and multivariate Cox analysis of the hub genes overexpressed in GBM patients. (a) OS of the hub genes overexpressed in GBM sufferers. (b) DFS of the hub genes overexpressed in GBM sufferers. (c) Univariate and multivariate Cox analysis of TIMP1 expression and other clinical pathological factors for OS. HR, hazard ratio.

OS and DFS analyses of the central genes were further conducted by using GEPIA database. As shown in Figure 8(a), high expressions of TIMP1, FN1, LOX, and POSTN in GBM patients were significantly correlated with worse OS. Adverse DFS were also significantly scanned in GBM patients with elevated TIMP1, FN1, LOX, and POSTN expression levels (Figure 8(b)). The results of log-rank *P* test showed that only the OS and DFS of TIMP1, FN1, LOX, and POSTN had statistical significance simultaneously.

Furthermore, univariate Cox regression analysis revealed that FN1 was remarkably correlated with the OS (HR 1.66401, 95% CI = 1.54102–1.79681, *P* < 0.0001), LOX was significantly correlated with the OS (HR 1.66184, 95% CI = 1.55921–1.77123, *P* < 0.0001), POSTN was observably correlated with the OS (HR 1.37421, 95% CI = 1.32001–1.43063, *P* < 0.0001), and TIMP1 was evidently correlated with the OS (HR 1.56064, 95% CI = 1.48016–1.64549, *P*< 0.0001). In addition, grade was remarkably correlated with the OS (HR 3.39671, 95% CI = 2.2964–5.02423, *P* < 0.0001) and radiation therapy was remarkably correlated with the OS (HR 2.05196, 95% CI = 1.18933–3.54026, *P* = 0.00979). Moreover, multivariate Cox regression analysis revealed that TIMP1 was an isolated risk factor for OS (HR 1.44208, 95% CI = 1.16537–1.7845, *P* = 0.00076). These results are summarized in Figure 8(c).

### 3.8. Immune Infiltration Analysis of GBM

**Figure 9:**
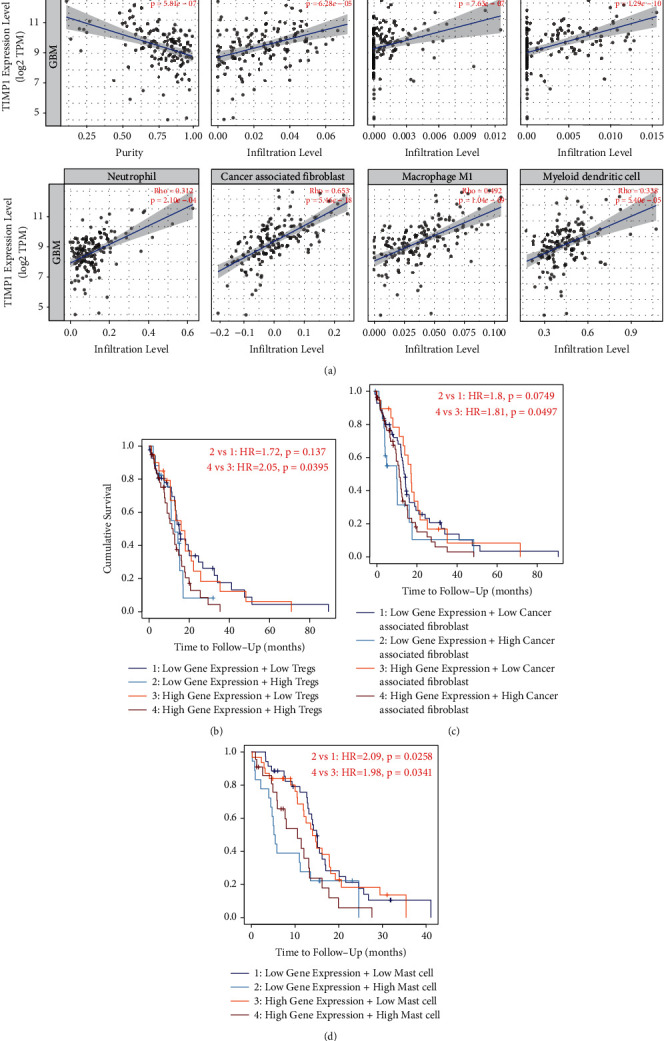
Correlation between immune infiltrates and TIMP1 expression in GBM. (a) TIMP1 expression was definitely connected with CD4+ T cell, B cell, T cell regulatory (Tregs), neutrophils, cancer-associated fibroblast, macrophage M1, and myeloid dendritic cell. (b–d) Higher infiltration of Tregs, cancer-associated fibroblast, and mast cell correlated with worse prognosis.

The degree of immune cell infiltration in tumor tissue is an isolated prognostic factor for the survival of different tumors. Therefore, we focused on analyzing the association between the expression of TIMP1 and infiltrating immune cells in GBM. TIMER2.0 revealed that the expression of TIMP1 was remarkably associated with the infiltration of CD4+ T cell (Rho = 0.335, *P* = 6.28*e* − 05), B cell (Rho = 0.408, *P* = 7.63*e* − 07), T cell regulatory (Tregs) (Rho = 0.514, *P* = 1.29*e* − 10), neutrophils (Rho = 0.312, *P* = 2.10*e* − 04), cancer-associated fibroblast (Rho = 0.653, *P* = 5.46e−18), macrophage M1 (Rho = 0.492, *P* = 1.04*e* − 09), and myeloid dendritic cell (Rho = 0.338, *P* = 5.40*e* − 05) in GBM. By comparison, it is negatively correlated with cancer purity (Rho = −0.41, *P* = 5.81*e* − 07) (Figure 9(a)).

To further explore the connection with GBM and the level of immune cell infiltration, we used TIMER2.0 to evaluate the connection with TIMP1 expression of various immune cells and immune marker genes in GBM. The results revealed that the expression level of TIMP1 was remarkably positively associated with most immune cell markers in GBM. Moreover, the result displayed that TAM marker (CCL2), macrophage M1 marker (PTGS2), macrophage M2 markers (CD163, VSIG4, and MS4A4A), neutrophil marker (ITGAM), and Treg marker (TGFB1) were observably positively associated with the expression level of TIMP1 in GBM (Table 4), which suggested that TIMP1 plays a vital role in the glioma immune microenvironment. Finally, higher Treg infiltration was related with worse prognosis for TIMP1 in GBM (Figure 9(b); HR = 2.05, *P* = 0.0395). Equally, higher cancer-associated fibroblast and mast cell infiltration was also related to poor outcome in GBM (Figures 9(c) and 9(d); HR = 1.81,*PP* = 0.0497; HR = 1.98, *P* = 0.0341).

### 3.9. TIMP1 Promotes GBM Cell Proliferation, Migration, and Invasion

**Figure 10:**
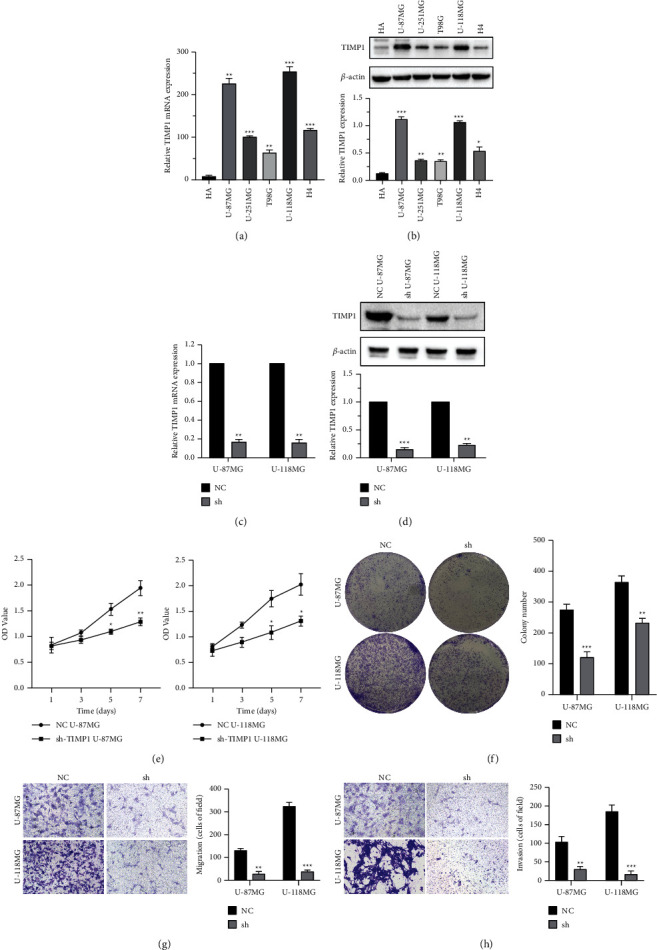
Downregulation of TIMP1 inhibits cell proliferation, migration, and invasion in U-87 MG and U-118 MG cells. (a, b) The results of qPCR and WB showed that compared with many glioblastoma cell lines, U-87 MG and U-118 MG cell lines were selected for TIMP1 gene related function test. (c, d) qPCR and WB showed that TIMP1 expression was inhibited in U-87 MG and U-118 MG cells transfected with sh-TIMP1 compared with the NC group. (e, f) CCK-8 and colony formation assays revealed that the proliferation abilities were suppressed in U-87 MG and U-118 MG cells transfected with sh-TIMP1 compared with the NC group. (g, h) Transwell assay revealed that cell migration and invasion were suppressed in U-87 MG and U-118 MG cells transfected with sh-TIMP1 compared with the NC group. ^*∗*^*P* < 0.05; ^*∗∗*^*P* < 0.01; ^*∗∗∗*^*P* < 0.001.

In order to further verify the role of TIMP1 in GBM, we firstly screened U-87 MG and U-118 MG cell lines with high expression of TIMP1 gene in multiple GBM cell lines by qPCR and WB experiments (Figures 10(a) and 10(b)). Second, U-87 MG and U-118 MG cell lines with TIMP1 knockdown were constructed by transfection of lentivirus sh-TIMP1, and the target gene knockdown efficiency of sh-TIMP1 U-87 MG and sh-TIMP1 U-118 MG cell lines was detected by qPCR and WB experiments. The results showed that the expression level of TIMP1 in the cell line transfected with sh-TIMP1 was significantly lower than that in the cells transfected with NC (Figures 10(c) and 10(d)). CCK-8 and colony formation experiments showed that downregulation of TIMP1 could significantly inhibit cell proliferation and clonality (Figures 10(e) and 10(f)). Transwell analysis showed that downregulation of TIMP1 significantly inhibited cell migration and invasion (Figures 10(g) and 10(h)).

## 4. Discussion

GBM is the most invasive and worst prognosis of high-grade gliomas (WHOIII and WHOIV gliomas). Although surgical resection, radiotherapy, and chemotherapy have achieved tremendous advance in modern medicine, the median survival time of patients after initial diagnosis is only 12–15 months. Difficult radical resection of gliomas and resistance to radiotherapy and chemotherapy are the main causes of recurrence and treatment failure [2, 3]. Early diagnoses will obviously enhance the clinical prognosis of GBM. Bioinformatics analysis can quickly and accurately identify biomarkers related to GBM development, which has important research value in the study of diagnostic markers and prognostic genes.

Bioinformatics is a subject that has developed rapidly in recent years by relying on big data processing, and it can extract valuable information for clinical research from massive data generated by multisource experiments [5, 22]. In this study, 5 gene expression profiles of GBM were retrieved from GEO and TCGA databases, including 343 glioblastoma specimens and 259 normal specimens. 54 common upregulated DEGs expression profiles and 22 common downregulated DEGs expression profiles were favorably identified, severally. The 10 central genes were selected according to the connectivity of PPIN, incorporating LOX, IGFBP3, CD44, TIMP1, FN1, VEGFA, POSTN, COL1A1, COL1A2, and COL3A1. Subsequently, we performed mRNA expression verification, gene change frequency, survival analysis, COX analysis, and tumor immune infiltration analysis of these 10 hub genes. Finally, four hub genes had been screened out including TIMP1, FN1, POSTN, and LOX. These identified central genes may play a crucial role in GBM.

FN1 encodes fibronectin, a glycoprotein that exists in the form of dimers or polymers on the cell surface and in the extracellular matrix [23]. POSTN encodes a protein that plays a significant role in tissue development and regeneration. These proteins are secreted to the extracellular matrix for tissue repair, such as granulation hyperplasia, mucosal repair, and ventricular remodeling [24]. LOX is a protein-coding gene that mainly encodes the lysyl-oxidase family and leads to a variety of transcriptional variants; one of the precoding proteins was hydrolyzed to produce a regulatory propeptide and a ripening enzyme [25].

TIMP1, also known as fibroblast collagenase inhibitor, is a protein-coding gene [26]. This gene was primarily known as an endogenous inhibitor of matrix metalloproteinases (MMPs) [27]. Among its related pathways are HIF-1 signaling pathway, IL6-mediated signaling events, and ECM organization. This gene family encodes proteins that are inhibitors of MMPs involved in extracellular matrix degradation [28]. With the exception of its suppressive role against most of the known MMPs, as a cytokine and a key regulator of ECM degradation, TIMP1 has a variety of functions related to tumor microenvironment and progression [29], tumor cell proliferation [30], and antiapoptotic activity in cancer [31–33]. Intriguingly, MMPs have been found to be synthesized mainly by adjacent and intervening stromal cells, similar to TIMP1 which is secreted in the tumor microenvironment [34, 35]. Similarly, we discovered that TIMP1 was negatively in connection with tumor purity through the TIMER2.0 database, which suggested that TIMP1 was mainly derived from stromal cells rather than GBM cells. A large number of research studies suggested that TIMP1 is often highly expressed in several types of human cancer cells, containing prostate cancer [36], lung cancer [37], melanoma [38], breast cancer [35], and glioblastoma [39]. Based on the above conclusions, we suggested that FN1, POSTN, LOX, and TIMP1 can be used for cancer diagnosis and as prognostic indicators.

Infiltration of immune cells into tumors is an essential factor affecting tumor occurrence and progression [40, 41]. For instance, Chen et al. proposed that the expression of CXCL10 is related to the infiltration of miscellaneous immune cells. Tumor-associated macrophages express CXCL10 in pancreatic cancer, and its acceptor CXCR3 is extremely expressed in T cells. Their study showed that the expression of CXCL10 was actively in connection with multiple immune core proteins and suggested that CXCL10 can be used as one of the available targets of immunotherapy [6].Therefore, we conducted immune correlation analysis of the four core genes (FN1, POSTN, LOX, and TIMP1). Analysis of the results about correlation between immune infiltrates and TIMP1 expression showed that TIMP1 involved in tumor immunity. The high expression of TIMP1 combined with the high proportion of Tregs, cancer-associated fibroblast, and MAST cell suggested a poor prognosis in GBM patients. On the other hand, we also detected that TIMP1 was particularly connected with the immune marker of tumor-associated macrophage, macrophage M1, macrophage M2, neutrophils, and Tregs. Hence, the above results confirmed that the value of TIMP1 as a gene diagnostic for GBM and the prognostic value of GBM patients with high TIMP1 expression are cheek by jowl connected with the immune microenvironment of GBM.

By constructing TIMP1 knockdown glioblastoma cell line, we further analyzed the role of TIMP1 in glioblastoma and found that TIMP1 promoted cell proliferation. Knocking down TIMP1 by lentivirus transfection inhibited cell migration and invasion. This may be one of the potential mechanisms of TIMP1 promoting cell proliferation.

The preprint of this paper was submitted in the early stage [42], and we added experimental verification later. In addition, the main limitations of this study are as follows: first of all, the expression levels of hub genes, such as TIMP1, POSTN, LOX, and FN1, have not been confirmed by immunohistochemistry in clinical specimens. Second, the role of these hub genes in the appearance and evolution of GBM is still vague and should be verified by *in vitro* and *in vivo* functional research studies.

## 5. Conclusion

In this study, we reported the results of a comprehensive bioinformatics analysis of biomarkers associated with glioblastoma prognosis and their association with immune-infiltrating cells. The wholesale data analysis in this research afforded a comprehensive bioinformatics analysis of DEGs that may be concerned in the progress of GBM. The shortcoming of insufficient samples was overcome by using five open access databases for new joint analysis. However, the molecular mechanisms of TIMP1 in GBM still need to be further studied. In general, our experiments recommended the requirement to more discreetly score TIMP1 as a biomarker and therapeutic target, in order to develop the new antibodies for GBM exploration and prevention, with the hope of providing new ideas for clinical diagnosis and treatment of GBM patients and effectively improving sufferer prognosis.

## Figures and Tables

**Table 1:** The GBM patients vs. normal samples capacity of GEO datasets.

Datasets	Sample capacity	
	Tumor tissues	Normal tissues
GSE4290	81	23
GSE7696	80	4
GSE13276	5	5
GSE29796	14	20
TCGA	163	207
All	343	259

**Table 2:** The common DEGs of four gene expression profiles (adj. *P* value <0.05, |logFC|>2.0).

Common DEGs	Gene symbol
Upregulated DEGs	TOP2A; ASPM; NDC80; IGFBP2; RRM2; LTF; COL4A1; IGF2BP3; PDPN; MEOX2; SHOX2; COL3A1; SOX4; COL1A1; CDK1; NAMPT; COL1A2; CHI3L1; VEGFA; LOX; IGFBP3; CD44; COL4A2; NES; POSTN; EZH2; TNC; ANXA1; TGFBI; PTX3; SOX11; CFI; BTN3A2; NID1; KIAA0101; ADAM12; HAS2; KLHDC8A; ANXA2; FN1; EMP1; VIM; MGP; TIMP1; PHLDA1; CENPU; CKS2; ENPEP; COL6A2; LAMC1; SMC4; BUB1B; C1RTGIF1

Downregulated DEGs	AK5; KCNK1; SNCA; GRM3; PEX5L; MOBP; STXBP6; RAB40 B; SPOCK3; DLG2; MAL; CNTNAP2; REPS2; SH3GL3; TPPP; ANK3; MBP; ZNF365; ATP8A1; PAIP2B; SEC14L5; SEPTIN4

DEGs, differentially expressed genes.

**Table 3:** Top ten hub genes with higher degree of connectivity.

Gene symbol	Gene description	Degree
FN1	Fibronectin 1	25
TIMP1	TIMP metallopeptidase inhibitor 1	21
CD44	CD44 molecule (Indian blood group)	21
COL1A1	Collagen type I alpha 1 chain	20
VEGFA	Vascular endothelial growth factor A	20
COL1A2	Collagen type I alpha 2 chain	18
POSTN	Periostin	16
COL3A1	Collagen type III alpha 1 chain	15
LOX	Lysyl oxidase	14
IGFBP3	Insulin-like growth factor binding protein 3	14

**Table 4:** Correlation analysis between TIMP1 and related genes and markers of immune cells in TIMER.

Description	Gene markers	GBM			
		None		Purity	
		Cor	*P*	Cor	*P*
CD8+ T cell	CD8A	0.093	0.251	−0.009	0.915
	CD8B	0.168	0.038	0.069	0.420
T cell (general)	CD3D	0.275	*∗∗*	0.088	0.308
	CD3E	0.266	*∗∗*	0.121	0.160
	CD2	0.273	*∗∗*	0.092	0.285
B cell	CD19	0.043	0.600	−0.026	0.760
	CD79 A	−0.037	0.654	−0.087	0.313
Monocyte	CD86	0.313	*∗∗∗*	0.111	0.198
	CSF1R	0.359	*∗∗∗*	0.166	0.053
TAM	CCL2	0.579	*∗∗∗*	0.490	*∗∗∗*
	CD68	0.406	*∗∗∗*	0.210	0.014
	IL10	0.397	*∗∗∗*	0.214	0.012
Macrophage M1	NOS2	0.185	0.022	0.266	*∗*
	IRF5	0.232	*∗*	−0.005	0.954
	PTGS2	0.421	*∗∗∗*	0.330	*∗∗∗*
Macrophage M2	CD163	0.639	*∗∗∗*	0.556	*∗∗∗*
	VSIG4	0.470	*∗∗∗*	0.316	*∗∗*
	MS4A4A	0.498	*∗∗∗*	0.380	*∗∗∗*
Neutrophils	CEACAM8	−0.049	0.549	−0.086	0.315
	ITGAM	0.447	*∗∗∗*	0.279	*∗∗*
	CCR7	0.275	*∗∗*	0.149	0.081
Natural killer cell	KIR2DL1	−0.007	0.929	−0.037	0.668
	KIR2DL3	−0.020	0.803	−0.083	0.335
	KIR2DL4	0.140	0.083	0.072	0.401
	KIR3DL1	0.036	0.656	0.023	0.793
	KIR3DL2	0.115	0.158	0.115	0.180
	KIR3DL3	0.034	0.675	−0.007	0.935
	KIR2DS4	0.174	0.032	0.136	0.112
Dendritic cell	HLA-DPB1	0.316	*∗∗∗*	0.175	0.041
	HLA-DQB1	0.241	*∗*	0.102	0.234
	HLA-DRA	0.339	*∗∗∗*	0.167	0.051
	HLA-DPA1	0.223	*∗*	0.100	0.245
Treg	STAT5B	−0.226	*∗*	−0.132	0.124
	TGFB1	0.411	*∗∗∗*	0.268	*∗*

^*∗*^ *P* < 0.01; ^*∗∗*^*P* < 0.001; ^*∗∗∗*^*P* < 0.0001.

## Data Availability

All the experimental data involved in this study are obtained from open source. Please refer to the materials and methods in this paper for specific access.
